# When healers get wounded! Moral injury in healthcare providers during the COVID-19 pandemic in Pakistan

**DOI:** 10.3389/fpsyt.2023.1244055

**Published:** 2023-09-19

**Authors:** Madah Fatima, Nazish Imran, Irum Aamer, Somia Iqtadar, Bilquis Shabbir

**Affiliations:** ^1^Academic Department of Psychiatry and Behavioral Sciences, Mayo Hospital, King Edward Medical University, Lahore, Pakistan; ^2^Child and Family Psychiatry Department, Mayo Hospital, King Edward Medical University, Lahore, Pakistan; ^3^Department of Medicine, Mayo Hospital, King Edward Medical University, Lahore, Pakistan; ^4^Department of Medicine, Sir Ganga Ram Hospital, Fatima Jinnah Medical University, Lahore, Pakistan

**Keywords:** moral injury, Covid-19 pandemic, potentially morally injurious events, SARS-CoV 2 pandemic, healthcare providers, Pakistan

## Abstract

**Introduction:**

Moral injury (MI) is a multi-faceted and multidimensional phenomenon. Occupational MI has been studied mainly among military personnel and first responders and is linked to mental health problems. MI encompasses negative moral emotions such as shame, guilt, and anger leading to distress, and impairment in social and occupational functioning. The COVID-19 pandemic predisposed healthcare providers to moral dilemmas, potentially morally injurious events (PMIEs), and MI. We aimed to assess the prevalence and predictors of MI in healthcare providers during the COVID-19 pandemic in Pakistan.

**Materials and methods:**

This cross-sectional study was conducted in July–October 2021 among physician/clinician staff working at teaching hospitals in Lahore. The Moral Injury Symptoms Scale-Health Professionals (MISS-HP) was used to collect data. SPSS 26 was used for data analysis applying Wilcoxon Mann–Whitney U and Kruskal–Wallis tests on non-normally distributed data at α = 0.05. Predictors of MI were ascertained through Binary Logistic Regression analysis.

**Results:**

Four hundred and twenty physicians responded to the questionnaires. The Median (IQR) MI scores were 37(28–47). Guilt, moral concerns, and shame were higher-scoring MI dimensions. 40.8% (*n* = 171) suffered from clinically significant distress and impaired functioning while 14.3% (*n* = 60) from severe distress. Gender, department, and history of psychiatric illness predicted higher levels of distress which were 1.9 times higher in females than males and 2.5 times higher with a history of psychiatric illness. Working on the front lines did not predict MI.

**Conclusion:**

Our findings highlight the substantial burden of MI in our sample during COVID-19, having implications for healthcare providers’ well-being, healthcare quality, and service delivery. This calls for concerted efforts from all stakeholders to better prepare for future disasters through effective human-resource policies, pre-trauma exposure soft-skills training, effective teamwork and communication strategies; self-stewardship and resilience modules, and mental health support for healthcare providers. The dimensional construct of MI may vary across cultures; hence we recommend further cross-cultural research on MI in healthcare providers, particularly in the context of public health disasters.

## 1. Introduction

The COVID-19 pandemic created an unprecedented and challenging situation for healthcare systems worldwide, from high-income countries to resource-constrained lower-middle, and low-income countries. The pandemic resulted in a combat environment for front-line healthcare workers in the battle against the novel coronavirus (1). Moreover, the inconsistencies in the implementation of medical and public health ethics compounded moral dilemmas for frontline healthcare providers (2). There was a grueling impact of the lack of “standard operating procedures,” improper implementation of mostly ineffectual state policies, and irresponsible public attitudes towards preventive measures on Pakistan’s already overstretched public health infrastructure (3). In addition, the imbalance between demand and supply of essential resources due to the sudden surge and ever-increasing number of cases of COVID-19 pneumonia preordained the delivery of low-quality healthcare services and the inexorable ethical issues faced by the frontline staff (4).

Potentially morally injurious events (PMIEs) occur in high-stakes situations as committing “morally wrong” actions and inactions or witnessing others’ acts of omission and commission, that may violate one’s long-standing and deeply ingrained moral values, behaviors, and expectations (5). Moral injury (MI) is characterized by negative thinking such as self-blame, resentment towards others, and negative emotions like shame, guilt, disgust and anger, leading to problems with social and occupational functioning (6). This form of psychological trauma may also result in untoward and long-term psychological, behavioral, emotional, spiritual, and social outcomes (7).

MI is not solely an occupational issue but a multifaceted and multidimensional phenomenon involving philosophical, social, existential, religious, and political dimensions. However, certain occupations may increase the risk of MI due to high stakes and morally stressful situations (8). Jonathan Shay, an American psychiatrist who coined the term in the 1990s while working with Vietnam War veterans, stated that MI occurs when either oneself or those in positions of power and authority “fail to do what is considered right” (9). The concept garnered the attention of clinicians and researchers long after the Afghanistan and Iraq wars and has since been extensively studied among war veterans and first responders such as police and civil defense officials worldwide (10, 11).

There has been limited discourse and research on MI in healthcare providers. Moral stressors, also phrased in the available literature as PMIEs became inevitable in healthcare settings during the pandemic. Decisions to withdraw a patient from a life-saving treatment *viz.* mechanical ventilation to make it available for other patients constitute PMIEs from one’s actions. PMIEs from inaction include failure to timely screening a patient, reaching a critical life-saving decision, delaying patient care for donning protective gear, and working at low patient-safety margins after being deployed to the COVID-19 high dependency units (HDUs) and intensive care units (ICUs) without prior training and skillset in critical care (5, 12). Fear of contracting the infection and infecting one’s family among clinicians could also affect the quality of patient care, creating moral distress (13). Healthcare providers having to shoulder the burden of triage decision-making and rationing of limited resources such as hospital and ICU beds among critical patients, administrative and policy decisions requiring tradeoffs between suboptimal care for individual patients and the larger interest of the community are a few examples of PMIEs related to witnessing others’ morally wrong actions or inactions (5).

The risk of MI in essential workers may increase with the lack of social support, irresponsible leadership, unsupportive management, and workers’ lack of awareness and preparedness to handle exposure to PMIEs and moral distress (14). Additionally, moral distress and injury are centered on trust: a set of beliefs, mental attitudes, and behaviors that encompass confidence in and reliance on oneself, others, and the world. Moral transgressions at work due to avoidable moral stressors may result in the breach of trust in one’s colleagues, the organization, and the overall system. This aspect makes occupational MI relational which may result in a sense of alienation and loss of meaningful relationships at work (15). Recent research has revealed a high prevalence of psychological distress including sleep disturbances, burnout, anxiety, depression, post-traumatic stress disorder, suicide, and substance use among healthcare workers during the COVID-19 pandemic (16, 17). Furthermore, research showed that the healthcare providers continued to have problems with psychosocial functioning including engagement with work and relationships, spirituality and self-care over the longitudinal course of 10 months after exposure to COVID-19-related PMIEs (18).

Limited original research has been conducted on MI among healthcare providers including clinicians across the world, particularly in Pakistan. The present study aims to explore the prevalence of MI and various factors contributing to it in healthcare providers (clinicians/physician staff) during the pandemic. The findings of this study will be helpful in devising future public health policies to address the psychosocial well-being of healthcare providers in the context of disaster preparedness and management. This study also highlights the significance of incorporating MI related discourse and research pertaining to the occupational health of healthcare providers, as public health disasters primarily increase the risk of MI, related distress, and impaired functioning in essential healthcare workers.

## 2. Materials and methods

This research was conducted in accordance with the general ethical guidelines of the Declaration of Helsinki. The Institutional Review Board (IRB) of King Edward Medical University (KEMU), Lahore approved the study proposal vide Letter no. 538/ARA/KEMU dated 10/07/2021. A cross-sectional study was conducted among healthcare providers (physician/clinician staff) working at teaching hospitals in Lahore, from July to October 2021 during the 4th wave of the COVID-19 outbreak in Pakistan. Physicians/clinicians aged 20–60 years including house officers (interns), medical officers (clinicians working on non-training posts), postgraduate residents, and all levels of clinical teaching faculty members including Senior Registrars, Assistant Professors, Associate Professors, and Professors were included. Those working for less than 3 months at the respective hospital were excluded. The non-probability convenient sampling technique was applied to recruit informants. Participants signed written informed consent before responding through paper-based questionnaires. The online forms containing a consent statement were circulated through social media mainly Facebook and WhatsApp groups. We made an introductory statement in the data collection forms regarding the ongoing discourse of moral distress and injury among healthcare providers during the COVID-19 pandemic and asked the respondents to focus on their MI related lived experiences since the onset of SARS-CoV2 pandemic in the country.

The 10-item Moral Injury Symptoms Scale-Health Professionals (MISS-HP) was used for data collection. MISS-HP is a reliable and valid tool that assesses ten dimensions including both psychological and religious aspects of MI in healthcare providers: betrayal, guilt, shame, moral concerns, loss of trust, loss of meaning, difficulty forgiving oneself and others, self-condemnation, struggles with faith, and loss of religious faith. Response options for each item range from 1 to 10 indicating the level of agreement with statements (1 = Strongly Disagree and 10 = Strongly Agree). The total score ranges between 10 and 100, with higher scores indicating greater severity of MI. MI symptoms associated with clinically significant distress and impairment in functioning can be detected at a sensitivity of 84% and specificity of 93% at MISS-HP scores equal to or above 36. Moderate to severe MI related distress and impaired functioning at work, in relationships, and in other areas of life is indicative of clinical significance and is measured through a statement having a 5-point Likert Scale: Not at all, Mild, Moderate, Very Much, and Extremely. “Not at all” and “Mild” denote clinically insignificant distress whereas “Moderate,” “Very Much” and “Extremely” indicate clinically significant distress and impairment in functioning (19).

Data analysis was done *via* IBM SPSS version 26. The positively worded items on MISS-HP were reverse-coded. The variable “MI related distress and impairment in functioning” was recoded into a dichotomous variable and the options Moderate, Very Much, and Extremely were consolidated into “Clinically Significant Distress.” Qualitative variables including background characteristics and severity of MI related distress were presented as percentage and frequency. The total and subscale MI scores were reported as Median and Interquartile Range (IQR). The non-parametric Wilcoxon Mann–Whitney U and Kruskal–Wallis tests were applied to the non-normally distributed data. Predictors of MI were ascertained through Binary Logistic Regression analysis. The level of significance was set at α = 0.05.

## 3. Results

### 3.1. Background characteristics

Four hundred and twenty healthcare providers participated in the study. The Median (IQR) age of the participants was 30 (28–34). 215 (51.2%) respondents were males whereas 205 (48.8%) were females. Nearly two-thirds (71.2%) were from medicine and allied departments which included General Medicine, Emergency Medicine, Cardiology, Pulmonology, Psychiatry, Neurology, Oncology, Dermatology, and Pediatrics. Rest were from Surgery and allied departments (including General Surgery, Anesthesia, Pediatric Surgery, Cardiothoracic Surgery, Plastic Surgery, Orthopedic Surgery, Neurosurgery). 54.3% had less than 5 years of professional experience and about two-thirds (76.9%) had provided direct care to COVID-19 patients. 10% had a history of psychiatric illness (Table 1). The history of psychiatric illness was operationalized through a question/statement asking if the respondent had ever suffered from mental health issues that interfered with their social and occupational functioning making them seek professional help or mental health consultation, formally diagnosed with a psychiatric illness, and/or treated pharmacologically or otherwise.

**Table 1:** Background features of the study participants.

Background Characteristics	Median	IQR
Age	30	28–34
	***f***	**%**
**Gender**		
Male	215	51.2%
Female	205	48.8%
**Marital status**		
Single	141	33.5%
Married	275	65.5%
Separated/Divorced	2	0.5%
Prefer not to say	2	0.5%
**Seniority level**		
Junior staff (House Officer, Medical Officer, Postgraduate Resident)	307	73.1%
Senior staff (Senior Registrar, Assistant Prof, Associate Prof, Professor)	113	26.9%
**Frontline or second line staff**		
Frontline	323	76.9%
Second line	97	23.1%
**Department**		
Medicine and Allied Department	299	71.2%
Surgical and Allied Department	121	28.8%
**Total professional experience**		
Less than 5 years	228	54.3%
5–10 years	99	23.6%
More than 10 years	93	22.1%
**History of psychiatric illness**		
Present	42	10%
Not present	378	90%

^†^IQR, inter quartile ratio.

### 3.2. Moral injury

The total Median (IQR) MI scores were 37 (28–47). Guilt and “moral concerns” were the highest-scoring subdimensions followed by “shame” and “loss of trust.” We found comparatively lower scores of “loss of meaning,” “self-condemnation,” “struggles with faith” and “loss of religious faith” (Table 2). Respondents from Surgery and allied departments including anesthesia, females, junior doctors, those having <5 years of experience, and those who worked on the frontlines and had psychiatric history scored higher (Table 3).

**Table 2:** Moral injury symptoms scale and subscale scores.

Total moral injury symptoms scale scores	Median (IQR)	Mean (SD)
	37 (28–47)	37.7 ± 14.5
**Moral injury subscale scores**		
*Sense of betrayal*I feel betrayed by other health professionals whom I once trusted	3 (1–6)	3.98 ± 2.9
*Guilt*I feel guilt over failing to save someone from being seriously injured or dying	6 (3–9)	5.76 ± 3.1
*Shame*I feel ashamed about what I’ve done or not done when providing care to my patients.	4 (2–7)	4.34 ± 2.9
*Moral concerns*I am troubled by having acted in ways that violated my own morals or values.	5 (2–7)	4.7 ± 2.9
*Loss of trust*Most people with whom I work as a health professional are trustworthy	4 (2–6)	3.98 ± 2.4
*Loss of meaning*I have a good sense of what makes my life meaningful as a health professional	2 (1–4)	2.75 ± 1.9
*Difficulty forgiving*I have forgiven myself for what’s happened to me or to others whom I have cared for.	3 (1–4)	2.99 ± 2.0
*Self-condemnation*All in all, I am inclined to feel that I’m a failure in my work as a health professional.	2 (1–4)	3.07 ± 2.5
*Struggles with faith*I sometimes feel God is punishing me for what I’ve done or not done while caring for patients.	2 (1–4)	2.83 ± 2.5
*Loss of religious/spiritual faith*Compared to before I went through these experiences, my religious/spiritual faith has strengthened.	2 (1–4)	3.21 ± 2.45

^†^IQR, inter quartile ratio.

**Table 3:** Total moral injury scores on MISS-HP against background characteristics.

Background characteristics	Median (IQR)	*p* value
*Gender*		
Female	39 (28–52)	0.02*
Male	37 (28–44)	
*Marital status*		
Single	39 (30–52)	0.02*
Currently married	37 (26–46)	
Divorced/separated	30.5 (27–34)	
Prefer not to say	25 (19–31)	
*Department*		
Medicine and allied	36.5 (26–46)	0.004*
Surgery and allied	41 (32–40)	
*Number of years in experience*		
<5 years	40 (31–52)	<0.001*
5–10 years	37 (26–46)	
>10 years	31 (19–40)	
*Level of seniority*		
Junior staff member	39.9 (30–51)	<0.001*
Senior staff member	31 (19–41)	
*Frontline versus second line staff*		
Frontline staff	38 (29–49)	<0.001*
Second line staff	33 (19–43)	
*History of psychiatric illness*		
Present	40 (35–55)	0.01*
Absent	37 (27–47)	

^*^p < 0.05: statistically significant; ^†^IQR, inter quartile ratio.

### 3.3. Moral injury-related clinically significant distress and impaired functioning

40.7% (*N* = 171) of respondents had MI related clinically significant distress and impaired functioning, 14.3% of whom suffered from the highest level of severity (Table 4).

**Table 4:** Moral injury related distress and impaired functioning severity levels.

Severity level	*f*	%		*f*	%
No to mild	249	59.3%	Clinically insignificant distress	249	59.3%
Moderate	111	26.4%	Clinically significant distress	171	40.7%
Severe	60	14.3%			

^†^f, frequency; ^‡^%, percentage.

### 3.4. Predictors of moral injury

Gender, department, and history of psychiatric illness were major predictors of MI related clinical distress and functioning impairment, which was 1.9 times higher in females than males and 2.5 times higher in those with a history of psychiatric illness (Table 5).

**Table 5:** Predictors of moral injury-related clinically significant distress and impaired functioning.

Variable	No. of cases/total respondents in category (%)	Adjusted OR [95% CI]	*p*
*Gender*			
Female	99/205 (48.3%)	1.910 (1.254–2.908)	0.003*
Male	72/215 (33.5%)	1 [Reference]	
*Department*			
Medicine and allied	109/299 (36.5%)	0.530 (0.337–0.835)	0.006*
Surgery and allied	62/121 (51.2%)	1 [Reference]	
*Number of years in experience*			
<5 years	111/228 (48.7%)	1.471(0.685–3.159)	0.3
5–10 years	38/99 (38.4%)	1 [Reference]	
>5 years	22/93 (23.7%)	1 [Reference]	
*Level of seniority*			
Junior staff member	143/307 (46.6%)	1.993(0.963–4.127)	0.06
Senior staff member	28/113 (24.8%)	1 [Reference]	
*Frontline versus second line staff*			
Frontline staff	133/323 (41.2%)	1.102 (0.672–1.807)	0.7
Second line staff	38/97 (39.2%)	1 [Reference]	
*History of psychiatric illness*			
Present	26/42 (61.9%)	2.527 (1.264–5.052)	0.009*
Absent	145/378 (38.4%)	1 [Reference]	

^*^p < 0.05: statistically significant; ^†^OR, odds ratio; ^‡^CI, confidence interval.

## 4. Discussion

Moral awareness, moral distress, clinical burnout, and MI all exist on a continuum. Healthcare providers may suffer from MI, leading to clinical burnout characterized by emotional exhaustion, depersonalization and detachment from work, upon repeated and rampant exposure to PMIEs. Moral distress is unavoidable in healthcare; however, concerted efforts can be made to minimize the risk of MI and clinical burnout through administrative interventions, structural reforms; and leadership and peer support for the frontline staff (20).

In our sample, the total Median and IQR scores of MI among healthcare providers during the 4th COVID-19 wave were 37 (28–47), and Mean and Standard Deviation scores were 37.7 ± 14.5, comparable to the Mean (SD) scores of 32.31 (13.26) reported in a study on German and 36.8 (13.3) among the US health professionals during the first wave of the Pandemic (19, 21). Moral concerns; and moral emotions such as guilt and shame, a sense of betrayal, and loss of trust were the higher-scoring subdimensions in our study. These results tie well with the experiences of the United Kingdom NHS health professionals that constituted a sense of betrayal and feeling of being ‘dehumanized’ by the local government, trust management and healthcare leadership during the COVID-19 pandemic. These concerns were mainly centered on a sense of loss of autonomy in patient care, and the ethically questionable decisions imposed on the healthcare providers by the leadership, hence curtailing the process of moral repair (22). Literature reveals that healthcare providers initially experienced fear of stigma, contracting the infection and transmitting it to family and vulnerable patients followed by a sense of alienation from patients and their families, and betrayal by coworkers and management during the COVID-19 pandemic (23).

In comparison to the findings of our study, in which the most eminent moral emotions were guilt and shame, the United Kingdom health professionals reported feeling angry, frustrated, and helpless during the pandemic (22). The feelings of guilt and shame among healthcare providers were driven by the assumptions of personal responsibility to compensate for systemic loopholes in the underfunded and under-resourced healthcare system. Being forced to work beyond competencies and expertise to sometimes provide substandard and poor-quality care and being put at the disposal of morally challenging situations due to organizational issues added to the psychological burden among healthcare providers during the pandemic (24).

Our results illustrated higher scores on the subdimensions of moral concerns and negative moral emotions such as guilt and shame; sense of betrayal and loss of trust. Ethical concerns and moral emotions indicate moral values and their expression, which may vary across sociocultural dynamics and may also be suggestive of individual factors related to moral resilience. We also found comparatively lower scores on difficulty forgiving oneself and others, struggles with faith, and loss of religious faith which are indicative of the religious and spiritual dimensions. Notably, higher ethical concerns and weaker spiritual beliefs have been linked to less moral resilience thus predicting MI related psychological distress in healthcare providers during the pandemic (25, 26). Forgiveness in the context of morally wounded healthcare providers implies handling anger towards oneself and others effectively and minimizing negative self-attributions and views of others. Various forgiveness interventions may be incorporated in psychological trauma care to restore positive cognitions, improve psychological and emotional well-being, and heal the sufferer’s relationship with oneself and others (27).

The prevalence of MI among health professionals ranged between 13.8 and 45.6% in the first waves of the pandemic across several high to upper-middle-income countries (25, 27–29). The prevalence of moderate to severe MI related distress and impaired functioning in our sample even during the fourth wave was as high and comparable as 40.7% with 14.3% having severe levels. In our study, females, junior participants, those having less than 5 years of experience, who provided direct COVID-19 care or had a history of psychiatric illness had higher total MI scores. A study from China conducted during the first wave of the pandemic also found that female gender, direct COVID-19 care, and junior-level staff positions were associated with higher levels of MI (27). Contrary to our results that showed higher MI scores in respondents with less than 5 years of experience, a study from the US mid-Atlantic region reported higher levels in those having more than 20 years of experience. Previous research highlighted that the participants with decades of experience had decreased reserves of moral resilience and higher levels of MI (25). Another US-based study reached a conclusion similar to ours in which younger age and lesser professional experience predicted MI (26).

We found that female gender, working in surgery and allied departments and history of psychiatric illness were major predictors of clinically significant distress and impaired functioning. Working on the frontlines or providing care to COVID-19 patients did not predict the severity of MI related distress in our study. A Canadian study correlated low moral resilience reserves and a high risk of MI related negative consequences with female gender and a history of psychiatric illness and also illustrated that moral resilience was a moderator between exposure to PMIEs and moral distress. However, direct care of COVID-19 patients was linked to higher levels of moral distress, anxiety, and depression, in contrast to our findings that frontline work did not predict higher MI related distress (30). Rushton and others also underscored that moral resilience was a stronger predictor and moderator of the negative sequelae of MI than working on the front lines (25).

The concept of low moral resilience uncoils the individual aspects that may make vulnerable staff more prone to the negative consequences of exposure to morally distressing situations. However, the protective resilience factors did not remain significant over a longitudinal course of 3 months since the onset of COVID-19, as elucidated by previous research (31). Healthcare providers who committed medical errors or experienced severe clinical burnout had higher odds of suffering MI (26). The “second victim” experience after being involved in patient harm was more prevalent during the pandemic compared with pre-pandemic times, in the presence or absence of clinically significant burnout syndrome. MI predicted clinical burnout during the pandemic (32).

MI related emotional disturbances may have serious psychiatric implications, resulting in depression, anxiety, PTSD, and suicide among healthcare providers (27, 33, 34). In previous research, the estimated risk of depression during the pandemic was 4.2 times and that of anxiety 3.8 times higher in the healthcare providers who experienced MI in comparison to the general population (27). Moreover, suicidal ideations were around three times higher in healthcare workers who had experienced psychological trauma and MI during the pandemic (35).

Previous research on MI among war veterans, military healthcare providers, first responders such as police servicemen, and essential workers during the COVID-19 pandemic indicates that MI is to be viewed as an ethical and social justice issue, as well as an occupational hazard (11, 36, 37). The non-conducive environment and psychological stressors at the workplace predicted high severity of MI among healthcare providers in a longitudinal course during the pandemic (31). The sense of personal safety and security, the right to rest and adequate sleep, flexible work hours and human resource policies, monetary benefits according to workload, administrative support, and protection from infection were various unmet fundamental human needs linked to MI in healthcare providers during the pandemic (13, 35).

Healthcare providers around the globe attribute chronic organizational and management issues such as underfunding and understaffing to their incessant struggles with moral distress and its adverse outcomes during the pandemic, therefore calling for systemic reforms (22, 24, 25). The organizational culture lacking ethical decision-making processes at all levels of management subjects the workers to high psychological and emotional demands through lack of rewards, poor social support, and substandard quality of work (29). It is imperative to follow a structured approach to difficult decision-making in times of crisis to ensure transparency, clear communication with the frontline staff, and their utmost support in carrying out such decisions (12). Moreover, long-term systemic reforms and organizational changes are required to ensure the well-being of healthcare providers during times of disaster (24).

Healthcare providers also need to establish a balance between their well-being and their duty to healthcare. Self-care and self-stewardship practices in challenging times may help healthcare providers avoid MI and its long-term adverse consequences through skillful management of their well-being and health (38). High-quality and accurate communication with the frontline staff about the expected nature of work and its physical and mental health consequences; discussions about PMIEs, critical incidents, and patient safety issues; “Schwartz-centered rounds” to reflect on the emotional aspects of work, and “buddy system” to pair the experienced and inexperienced team members for direct supervision and support are a few of many effective communication and teamwork strategies (39). Early screening and identification of the struggling staff, ongoing monitoring of those exposed to PMIEs, informal support through a sense of solidarity and camaraderie among the trauma-informed and trained staff, and formal support through specialist consultations, cognitive behavioral and trauma-focused Therapies are the administrative responsibilities (40).

Self-care is pertinent to function optimally during such unprecedented times. Furthermore, mutual support and effective communication among team members may be helpful in processing negative emotions and managing stress. Hospital-level interventions constitute good supervision of the junior staff to incorporate positive values and attitudes towards patient-centered care, and psychological safety through the fulfillment of physical and financial needs, competency-building, and pre-trauma exposure training. Regular feedback mechanisms related to actions, policies, and initiatives of the administration and high-ups, and flexible redressal of healthcare providers’ needs may be effective in building trust in leadership. The systemic reforms and organizational changes such as focusing on funding, staffing, etc. would not occur overnight and require prioritization and collaborative efforts throughout the disaster preparedness cycle.

Minimal evidence in terms of original studies on MI in healthcare providers or clinicians is available from around the world (41). To our knowledge, this is one of the pioneer studies from Pakistan that addressed MI and ascertained its prevalence and predictors in healthcare providers during the COVID-19 pandemic to add to the body of research from around the world and inform all levels of policy decisions locally. We would also like to highlight a few caveats and limitations in our study. On account of the cross-sectional design of our study and lack of baseline MI levels prior to the pandemic and its longitudinal course afterward, it is difficult to attribute MI exclusively to the COVID-19 pandemic. Therefore, we recommend future studies to assess MI in healthcare providers in the absence of a crisis or a combat situation. We also recommend qualitative exploration of the lived experiences of healthcare providers with MI. The small convenient sample from tertiary care hospitals in one city also limits the generalizability of the results over clinicians or healthcare providers working across the country. We included only the clinician staff in our sample whereas MI may occur in any healthcare worker group involved in direct healthcare or any level of healthcare decision-making. In view of that, our study does not establish differences in MI among different staff positions. Therefore, we recommend the future studies to include and compare the phenomenon among multiple groups and staff positions including the nursing, administrative, and support staff, which was not an objective of our study. In our study, we used the original English version of MISS-HP. We recommend cultural adaptations of the scale in the local Urdu language to understand the phenomenon of occupational moral distress and injury in healthcare providers across socio-cultural dynamics.

## 5. Conclusion

The COVID-19 pandemic weighed heavily on the psychological and emotional well-being of healthcare providers including clinicians/physicians worldwide by exposing them to morally distressing situations, especially in the underfunded and resource-constrained healthcare systems of low-income and lower-middle-income countries. Many left their jobs in the health sector globally during the pandemic and others continued to suffer from ongoing psychological and emotional sequelae. This paper concludes that a considerable proportion of clinicians in our sample suffered from MI even during the fourth wave of the COVID-19 pandemic in Pakistan. Even though individual resilience and self-efficacy factors may help the service providers effectively process moral distress and negative emotions, MI has an occupational health dimension, deep-seated in systemic loopholes, that warrants organizational reforms, particularly the need for emphasizing the well-being of service providers as a crucial component of disaster preparedness and response. Moreover, we recommend further research into the subject to identify cross-cultural differences in MI and its dimensions among healthcare providers.

## Data availability statement

The raw data supporting the conclusions of this article will be made available by the authors, without undue reservation.

## Ethics statement

The studies involving humans were approved by Institutional Review Board of King Edward Medical University Lahore. The studies were conducted in accordance with the local legislation and institutional requirements. The participants provided their written informed consent to participate in this study.

## Author contributions

NI conceived the idea of the study and conceptualized the methodology with the help of IA, SI, and BS. NI and MF did a literature review, formal analysis, writing—preparation of tables, original draft preparation and editing, and final manuscript writing. NI, IA, BS, SI, and MF collected data and reviewed the manuscript critically. NI also supervised the whole project. All authors contributed to the article and approved the submitted version.

## Conflict of interest

The authors declare that the research was conducted in the absence of any commercial or financial relationships that could be construed as a potential conflict of interest.

## Publisher’s note

All claims expressed in this article are solely those of the authors and do not necessarily represent those of their affiliated organizations, or those of the publisher, the editors and the reviewers. Any product that may be evaluated in this article, or claim that may be made by its manufacturer, is not guaranteed or endorsed by the publisher.
